# The Encyclopedia of Proteome Dynamics: the KinoViewer

**DOI:** 10.1093/bioinformatics/bty823

**Published:** 2018-09-19

**Authors:** Alejandro Brenes, Angus I Lamond

**Affiliations:** Centre for Gene Regulation and Expression, School of Life Sciences, University of Dundee, Dundee, UK

## Abstract

**Summary:**

The Encyclopedia of Proteome Dynamics (EPD) ‘KinoViewer’ is an interactive data visualization tool designed for analysis and exploration of both protein and transcript data, showing expression of kinase genes in either human or mouse cells and tissues. The KinoViewer provides a comprehensive, updated graphical display of all human/mouse kinases and an open access analysis tool for the community with a user-friendly graphical interface.

**Availability and implementation:**

The KinoViewer is based on a manually drawn SVG, which is utilized with D3.js to create a dynamic visualization. It can be accessed at: https://peptracker.com/epd/analytics/. The KinoViewer is currently only accessible through the EPD, it is open access and can be used either to view internal datasets, or used to upload and visualize external user datasets.

**Supplementary information:**

Supplementary data are available at *Bioinformatics* online.

## 1 Introduction

Protein Kinases are a class of enzymes that catalyse the transfer of the gamma phosphate group from ATP onto specific hydroxyl groups on amino acid sidechains. The site-specific phosphorylation of protein substrates can drastically alter their function, by changing, for example, either their activity, interactions, localization and/or stability. As such, many protein kinases are key elements within signal transduction pathways and studying their expression and mode of action can be critical for characterizing mechanisms regulating such processes as development (Liu *et al.*, 2017), renewal (Annerén *et al.,* 2004; Kinehara *et al.,* 2013) and disease (Lahiry *et al.*, 2010). The protein kinase family is also of major clinical relevance and there are over 240 kinase inhibitors that are either already drugs, or else involved in clinical trials (Klaeger *et al.*, 2017), highlighting the interest in tools for quantitative analysis and visualization of kinases.

## 2 The KinoViewer

Following the completion of the draft human genome, the first detailed analysis of the human ‘Kinome’, i.e., the set of genes encoding protein kinase enzymes, was published over 15 years ago (Manning, 2002). This reported a total of 518 protein kinases within the human Kinome. However, this number has been revised in light of more recent data (Braconi and Orchard, 2008; The UniProt Consortium, 2017).

**Fig. 1.**
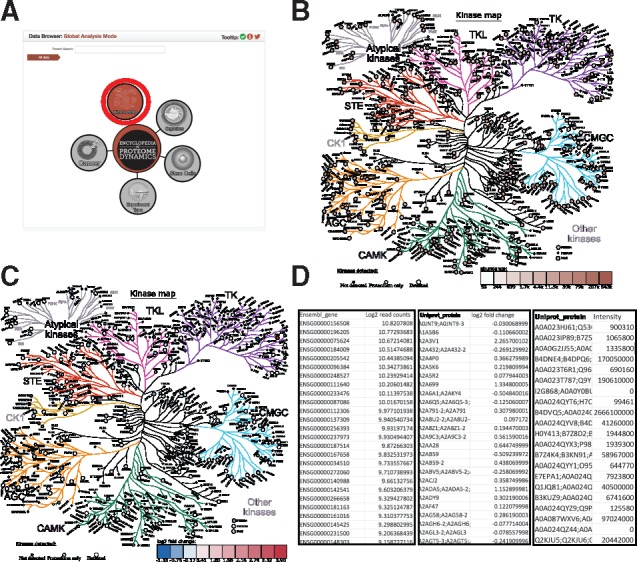
The Kinoviewer. (**A**) The graphical navigation: The KinoViewer access point from the EPD navigation (**B**) The KinoViewer – Copy numbers: Example of the KinoViewer visualization based on Copy number input. (**C**) The KinoViewer – log_2_ fold change: Example of the KinoViewer visualization based on log_2_ transformed ratios. (**D**) Example Data: Input data for the KinoViewer showing 3 different examples: transcript data mapped Ensembl Gene with log_2_ read counts, Protein data with fold change and protein data with intensity values

The Encyclopedia of Proteome Dynamics (EPD), is an open access, searchable online database (Brenes *et al.*, 2018). The KinoViewer was created as a scalable vector graphic diagram, which is integrated to a dynamic visualization via D3.js within the EPD. It is accessible at https://peptracker.com/epd/analytics through the graphical navigation by clicking on the red node labelled “Kinase Map”, as illustrated in Figure 1, panel A. The EPD KinoViewer presents a comprehensive list of the currently recognized human and mouse genes encoding protein kinases, organized as a phylogenetic tree, as shown in Figure 1, panels B & C (note branch lengths are not drawn to scale). It is implemented as an interactive visualization, supporting the display of quantitative data on the expression of protein kinase genes at either the protein, or transcript, levels. The KinoViewer design has updated the previously described protein kinase phylogenetic (Manning, 2002), both to remove elements that are no longer classified as Kinases, as well as to add additional protein kinases that were not originally included, e.g. PIK3CA and PIK3CG. Furthermore, the CMGC branch of the tree has been remodelled to represent an updated phylogenetic classification of the CDK family (Malumbre, 2014).

To visualize internal data within the EPD database, simply navigate through the EPD hierarchies, and click on the red KinoViewer node associated with any specific dataset. To use the KinoViewer to analyze user-provided data, tabular data describing kinase gene/protein expression must be uploaded as follows. The first column of the data is labelled with a header that describes the type of identifier used. The supported options are either ‘Uniprot_gene’, or ‘Uniprot_protein’, if dealing with proteomics datasets, and ‘Ensembl_gene’, or ‘Ensembl_transcript’, for transcriptomics datasets. ‘Uniprot_gene’ works with UniProt gene name for each protein kinase and ‘Uniprot_protein’ works with the corresponding UniProt accession. For transcriptomics data, the KinoViewer currently only accepts Ensembl identifiers. The second column should be used to provide quantitative data, if available, and has no labelling requirements for the header.

Example input data are illustrated in Figure 1, panel D and can be downloaded by clicking on the button labelled, ‘Download Example Data’ (more details about data formatting and transformations are available in the Supplementary Data section). Once correctly formatted data are pasted into the data input box labelled ‘Dataset input’, click the red button onscreen labeled ‘Submit’ to generate the visualization. The KinoViewer then updates to display all of the protein kinases that were detected within the uploaded dataset, showing these as larger circles, compared with the small grey circles for the kinases that were not detected.

As far as quantitative data types are concerned, the user can select any numeric category that is relevant for their analysis. For example, the KinoViewer can accept measures of *Abundance*, such as either protein copy numbers, for proteomics data and fragments per kilobase million (FPKM), for transcriptomics data (more details are provided in the Supplementary Data section). The KinoViewer will use these values to generate an interactive visualization on the protein kinase phylogenetic tree, displaying each detected protein kinase on its corresponding graphical element using a colour scale to represent the provided abundance value, as shown in Figure 1, panel C. In addition, to expand its utility for biological analyses, the KinoViewer also displays values that are comparisons between different experimental conditions. For example, values such as a Log_2_ transformation applied to a ratio comparing kinase gene expression under two specified conditions, or states (e.g. two different cell types, or time points, or +/- drug treatment etc). An example of the output from such a comparative analysis is shown in Figure 1, panel C. Note that for this type of comparative analysis the colour scale is modified from differential shading using a single colour, to a diverging colour scheme, to better represent the changes between conditions (further details are provided in the Supplementary Data section).

## 3 Conclusion

To meet the growing challenge of data analysis and visualization in the omics field, where experimental data sets are rapidly increasing in scale and complexity, we have focused on creating computational resources to make this process simpler, more intuitive and more powerful. The creation of the KinoViewer provides a good example of how analysis of complex, multi-omics data can be facilitated for researchers. Quantitative analysis on how the expression of kinase genes is regulated in different systems can now be visualized easily and conveniently on a dynamic, manually curated, and interactive representation. The resulting kinase expression maps can then be downloaded and used for presentations and further analysis. We expect the KinoViewer can thus become a valuable analysis tool for the research community.

## Funding

This work was supported by the Wellcome Trust [grant numbers 073980/Z/03/Z, 105024/Z/14/Z].

*Conflict of Interest:* Angus I Lamond is a cofounder of Platinum Informatics Ltd.

## Supplementary Material

Supplementary Data
