# Supplementary material for: The Encyclopedia of Proteome Dynamics: the KinoViewer
Source: Bioinformatics. 2018 Sep 19;35(8):1441–2. doi: 10.1093/bioinformatics/bty823 (PMC6477970; doi:10.1093/bioinformatics/bty823)
Supplement: Supplementary Data [file bty823_supp.zip › bty823-Suppl_data/bty823_supplemental_data.docx]

Supplemental Data

KINOVIEWER

The KinoViewer is a manually drawn Scalable Vector Graphic (SVG) created using Affinity Designer. The design features an updated classification of the CDK family to reflect information from recent phylogenetic studies (Malumbre, 2014). This SVG was then integrated within the Encyclopedia of Proteome Dynamics (EPD) (Brenes et al.,2018) JS ecosystem to create a dynamic visualisation with D3.js. The KinoViewer is powered by Neo4j where all the Kinase associations are created and modelled in a graph database.

The KinoViewer is accessible at <https://peptracker.com/epd/analytics/> by clicking on the red node with the label, ‘Kinase Map’

HUMAN AND MOUSE KINOME

The KinoViewer is currently implemented to work specifically with the human and mouse kinomes. If/when more kinase mapping files are provided by UniProt, the KinoViewer will also be updated to display these.

Based on information from the latest UniProt release (Braconi and Orchard , 2008;The UniProt Consortium, 2017), there are currently 506 genes encoding protein kinases within the Human Kinome and 507 in the Mouse Kinome.

MULTI-OMICS DATA INPUT

To use the EPD KinoViewer to visualise external user data, the data should be uploaded in tabular format and must conform to the following rules. The first column of the input data must contain the *header names*. If quantitative values are also available, these *data values* must be provided in the following column, as illustrated in Supplemental Figure 1 Panels A & B. The data can be pasted into the KinoViewer upload box as either Tab Separated Values (TSV), or Comma Separated Values (CSV). The data can thus contain either one, or two columns, depending on user preference, and the desired objective.

**Supplemental Figure 1:** A- Protein based input: The protein group column of a MaxQuant output and an intensity column. B- Ensembl Gene input: The Ensembl gene identifier output with Log_2_ Read Counts. C- The KinoViewer data input: Example of input data based on Gene name into the KinoViewer data input text area. The text area and the submit button are highlight with a red square.

As shown in Supplemental Figure 1, the first column of the data for upload must be labelled with a header that describes the type of identifier used. The supported options are either ‘Uniprot_gene’, or ‘Protein’, if dealing with proteomics datasets and ‘Ensembl_gene’, or ‘Ensembl_transcript’, for transcriptomics datasets. This column specifies the type of identifier that is used within the dataset. If used incorrectly, the result will be that the map returns empty. ‘Gene’ works with the UniProt gene name for each protein kinase. However, this has been adapted to work also with the Manning nomenclature (Manning et al., 2002). ‘Protein’ works with the corresponding UniProt accession. For transcriptomics data, the KinoViewer currently only accepts Ensembl identifiers.

The second column should be used to provide quantitative data, if available. Example input data can be downloaded by clicking on the button labelled ‘Download Example Data’ and is also provided as an excel file within the supplemental data.

The buttons illustrated in Supplemental Figure 1 Panel C regulate the functionality of the KinoViewer. To plot data after it has been pasted into the text area, click on the ‘submit’ button. This will initiate the analysis process, based on the input provided. If the header is not correctly formatted, a warning message will be displayed. Additionally, the 3 buttons on the upper right hand of the plot control the visibility of the protein kinase name annotations within the plot. Users can display the names of either only the subset of protein kinases that were detected in the dataset submitted, all protein kinase names, or else display no name labels at all. The EPD also provides a dynamic tooltip, which works with on-click actions, providing display of the names for the protein kinases and corresponding quantitative data, if available, specifically when clicking on any of the graphical elements that represent each of the individual protein kinases on the map.

QUANTITATIVE DATA

**Supplemental Figure 2: KinoViewer and quantitative data** A- Log_2_ Fold Change displayed on the KinoViewer. B- Copy number data displayed on the KinoViewer

The KinoViewer has been created to accept multiple types of user supplied quantitative data, along with the two most common types of data formatting. Either the ‘full point’ or ‘comma’ are both accepted as valid decimal separators. However, the input data cannot have delimiters used for readability, for example 100,000.02 would be invalid, the correct input would be either 100000.02 or 100000,02.

As far as input data types are concerned, the user can select any numeric category that is relevant for their analysis. For example, the KinoViewer can accept measures of *Abundance*, such as Protein Copy Numbers, for proteomics, and Fragments Per Kilobase Million (FPKM), for transcriptomics data. The KinoViewer will use these values to generate an interactive visualisation on the protein kinase phylogenetic tree, displaying each detected protein kinase on its corresponding graphical element using a colour scale based on the provided abundance value, as shown in Supplemental Figure 2, Panel B. In addition, to expand its utility for biological analyses, the KinoViewer also accepts measures of comparison between different experimental conditions. For example, values such as a Log_2_ transformation applied to a ratio comparing two specified conditions or states (e.g. two different cell types, or time points, or +/- drug treatment etc). An example of the output from such a comparative analysis is shown in Supplemental Figure 2, Panel A. Note that for this type of analysis the colour scale is modified from differential shading using a single colour, to a diverging two colour scheme, to better represent the changes between conditions.

QUANTITATIVE DATA LABELLING

The KinoViewer assumes the quantitative multi-omics data have not been previously transformed by a logarithmic function, common transformations include Log_10_ for protein abundance and Log_2_ for Reads or Ratios. The KinoViewer will then proceed to do the required transformations to optimise the colour scale presented to the user. If the data provided have already been transformed, label the column with the ‘Log10’ or ‘Log2’ prefix, for example ‘Log10 Copy Numbers’ or ‘Log2 Fold Change’.

This will provide the context the KinoViewer needs to deal with the input data, and prepare the colour scheme that will be best suited to display such data. Failure to label transformed columns with the appropriate headers will result in the data being transformed again.

PROTEIN GROUP HANDLING

If the identifier column has multiple candidate proteins/genes the KinoViewer will utilise the lead razor element, i.e. the first element in the group, as the candidate to be evaluated. This can mean that results that have been searched against TrEMBL will display less kinases than were actually detected in the dataset.

TrEMBL stands for Translated EMBL and it is a computer-annotated unreviewed supplement to Swiss-Prot. Unlike Swiss-Prot, TrEMBL has no manual review of the annotations, as such UniProt has not produced an official mapping file for kinases. It is therefore recommended that for datasets searched against the TrEMBL database, the results be copied utilising the UniProt Gene name.

REFERENCES

Braconi Quintaje, Q and Orchard S (2008) The annotation of both human and mouse kinomes in UniProtKB/Swiss-Prot: one small step in manual annotation, one giant leap for full comprehension of genomes. *Moll Cell Proteomics,* 7, 1409-1419

Brenes, A et al (2018) The Encyclopedia of Proteome Dynamics: A big data ecosystem for (prote)omics. *Nucleic Acids Res*., **46**, D1202-D1209.

Malumbre, M. (2014) Cyclin-dependent kinases. *Genome Biology*, **15**, gb4184.

Klaeger, S et al (2017) The target landscape of clinical kinase drugs. *Science*, **358**, eaan4368.

Manning, G et al (2002) The protein kinase complement of the human genome. *Science*, **298**, 1912-1934.

The UniProt Consortium. (2017) UniProt: the universal protein knowledgebase. *Nucleic Acids Res*, **45**, D158-D169.
